# Support for Employees with ASD in the Workplace Using a Bluetooth Skin Resistance Sensor–A Preliminary Study

**DOI:** 10.3390/s18103530

**Published:** 2018-10-19

**Authors:** Michał T. Tomczak, Marek Wójcikowski, Paulina Listewnik, Bogdan Pankiewicz, Daria Majchrowicz, Małgorzata Jędrzejewska-Szczerska

**Affiliations:** 1 Faculty of Management and Economics, Gdańsk University of Technology, 80-233 Gdańsk, Poland; michal.tomczak@pg.edu.pl; 2 Faculty of Electronics, Telecommunications and Informatics, Gdańsk University of Technology, 80-233 Gdańsk, Poland; paulist@o2.pl (P.L.); bogpanki@pg.edu.pl (B.P.)

**Keywords:** Autism Spectrum Disorders, employees, workplace, skin resistance sensor, Bluetooth sensor, Bluetooth

## Abstract

The application of a Bluetooth skin resistance sensor in assisting people with Autism Spectrum Disorders (ASD), in their day-to-day work, is presented in this paper. The design and construction of the device are discussed. The authors have considered the best placement of the sensor, on the body, to gain the most accurate readings of user stress levels, under various conditions. Trial tests were performed on a group of sixteen people to verify the correct functioning of the device. Resistance levels were compared to those from the reference system. The placement of the sensor has also been determined, based on wearer convenience. With the Bluetooth Low Energy block, users can be notified immediately about their abnormal stress levels via a smartphone application. This can help people with ASD, and those who work with them, to facilitate stress control and make necessary adjustments to their work environment.

## 1. Introduction

Workforce diversity is an issue that many researchers have taken into consideration, focusing on different dimensions of this phenomenon, e.g., age, race, gender, sexual orientation, culture and disability [1]. However, it is important to understand that diversity, in general, does not only bring positive consequences, e.g., an increase in performance, creativity, and innovation. The heterogeneity of teams can be the cause of intra-group cohesiveness reduction, and as a result, can lead to misunderstandings and conflicts [2].

Despite the fact that people with disabilities are a growing group among workers, in comparison to other diversity factors, disability has received relatively little research attention [3]. Unfortunately, there are not many strategies designed for overcoming work barriers caused by disabilities. Among existing implications, some can include diversity training, training for people with disabilities or dealing with co-workers’ resentment for overcoming individual barriers. There are also strategies for overcoming organizational barriers, such as supervisor training, mentoring/sponsoring programs, and accommodation [4], which is the most important strategy of the considered problem that our article refers to. Accommodation strategy can be defined as any reasonable adjustment in the workplace that allows a disabled person to increase work performance capacity. The adjustments can be provided by making facilities accessible and usable for disabled persons or by modifying equipment and devices [5].

Nowadays, there are only a few companies focused on recruiting neurodiverse employees [6], e.g., Towers Watson, E-Y, and Microsoft in the U.S. [7] or the mining company Weir Minerals, cloud computing firm Salesforce, Bankwest, and Hewlett Packard in Australia [8]. The software company SAP implemented the ‘Autism at Work’ program, integrating people with autism into the company’s workforce. The company’s objective is to make people with autism 1% of its global workforce, by 2020 [9]. Furthermore, the Danish social innovator company Specialisterne established a ‘gold standard’ of neurodiversity, with 75% of its workforce diagnosed with ASD [8].

Autism Spectrum Disorders (ASD) include autism and two other related disorders: Asperger Syndrome (AS) and pervasive developmental disorder-not otherwise specified (PDD-NOS) [10]. ASD is a category of developmental disorders, difficulties in social reciprocity, interpersonal communication, and unusual repetitive behavior [11]. People with ASD face difficulties with motor coordination resulting in an impairment of joint-action coordination, leading to failure in coordinating the movements with another person [12]. Other impairments that are characteristic for this group are poor imitation and joint attention [13,14,15], which is an ability to coordinate visual attention with another person and shifting the gaze toward a shared object [16]. As a result, few traits that strongly influence the situation of these people in the workplace, can be indicated. Firstly, they have difficulties with social interaction. Secondly, they have difficulties during the communication process (including verbal and nonverbal communication). Additionally, a lack of eye contact with the interlocutor makes building an interaction, even more difficult or impossible [17,18]. However, the most important problem for these people is the lack of ability to recognize stressful situations, which may result in reaction and behavior that is inadequate to the circumstances. As a result, adults with ASD are disadvantaged regarding employment [19]. They also experience a higher unemployment rate, not only in comparison to the general population but also in relation to adults with other disabilities [20]. On the other hand, people with ASD have a high interest in IT and a broad understanding of the functioning of electronic devices, which can be their strength in the contemporary (digitalized) workplace. Moreover, their advantages are also traits, such as sensitivity to details and patterns resulting from different cognitive styles [21] that can be successfully utilized in work positions within computer science professions, such as computer system administrators, analysts, testers and operators of telecommunications and IT systems, website programmers and administrators, database designers, and administrators or applied computer science specialists.

One of the main challenges in the future can be utilizing technology to facilitate or even enable disabled people to take up the work. Technological innovations have tremendous potential to assist both early diagnoses as well as intervention programs [22]. For years, research has been focused mostly on children with ASD, or on young adults in their 20s and 30s. There have been several studies focusing on an examination of the physiological response and engagement during sociocognitive tasks conducted by autistic children, using an electrocardiographic (ECG) signal-recording chest-belt, [23,24]. Other studies have investigated the neural correlates of response and initiation in children with ASD, using an integrated EEG/eye tracking system [15]. Existing sensing technologies for ASD screening and intervention can be categorized into eye trackers, movement trackers, electrodermal activity monitors, tactile sensors, vocal prosody and speech detectors, and sleep quality assessment devices [22]. Unfortunately, people in mid-to-late adulthood who are suffering from ASD were neglected as research subjects [19], and therefore, there is very little research that explores the experiences of adults, e.g., neurodiverse tech workers [21]. There are only a few tools (both hardware and software) that can be useful for this specific group, e.g., an iPhone application used for teaching targeted socio-vocational skills to adults with ASD [25], or compact wearable devices, such as “iCalm” for electrodermal activity (EDA), temperature, motor activity, and photoplethysmography (PPG) for long-term measurement [26]. Later, its commercially-available version “E4” wristband have also been made available, including a photoplethysmograph for the heart rate, 3-axis accelerometer for movements, and optical infrared thermometer for detecting skin temperature [27]. There is also a system of electronic sensors network measuring physiological parameters associated with emotional state changes to foster behavioral therapy for autistic children [28,29], or other IT solutions to improve therapeutic practices for children with ASD [30,31,32]. More importantly, to the authors’ best knowledge, none of these solutions has been implemented for adults, yet, so there is a strong need to provide tailored solutions for ASD employees.

There are, however, other wearable and non-wearable sensing devices for stress recognition that have been developed for the general population and not specifically for people with ASD, such as an intelligent emotion detection system for mobile phones that is implemented as a smart keyboard, which infers a user’s emotional state by a built-in accelerometer recording texting speed, time between presses, and shaking of the hand [33]. Another stress measuring system was based on features derived from smartphones and chest belts. It uses information from audio, physical activity, communication data collected, during the workday, and heart rate variability data collected at night, during sleep, to build multinomial logistic regression models [34]. There are also other systems using smartphones and wireless wearable sensors for detecting human activity [35,36,37], emotion recognition and classification [38,39] or stress monitoring e.g., “AMMON” [40], “MoodSense” [41],“StressSense” [42] and sensors based on the Heart Rate Variability [43,44]. We can also find more than a thousand commercially available smartphone applications for stress recognition. Unfortunately, the majority of them follow only the common approach of asking and providing a textual description on the ways of dealing with negative consequences of stress, and only some applications provide an additional possibility of tracking the behavior related to negative affect and stress [34].

The main motivation of the research presented in this paper is the idea that it is possible to develop sensors, wearable by employees with ASD, to indicate stressful situations, in the workplace, that is caused by a change of physical factors, as was described by Han et al. [45]. One of the most reliable stress indicators is a change in skin resistance [46,47,48]. Therefore, the authors designed and validated the Bluetooth skin resistance sensor. This solution will allow for the rapid recognition of physiological parameter changes, indicating increased stress levels [49,50,51,52]. As a result, it will allow for the notification of an increase in stress level, in real time, facilitate the ability to control stress, and make functioning in the work environment easier. Furthermore, applying for a job and then maintaining it will be less problematic for this group of people and the employment level among adults with Autism Spectrum Disorders (ASD) could increase.

## 2. Proposed Method

In order to monitor a stressful situation in the workplace, caused by a change in physical factors, in employees with ASD, a skin resistance sensor was selected.

### 2.1. Two-Point Skin Resistance Measurement

**Figure 1:**
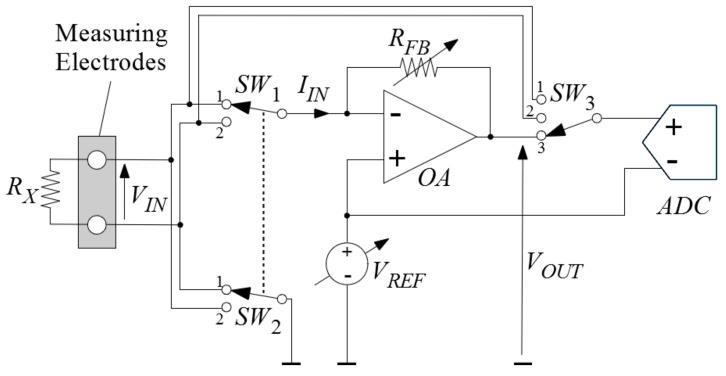
Basic schematic diagram of the resistance measuring circuit (*OA*—the operational amplifier, *V_REF_*—the programmable reference voltage source, *R_FB_*—the programmable resistor in the feedback loop, *R_X_*—the unknown resistance, *SW*_1_, *SW*_2_, *SW*_3_—the switches, *ADC*—the analog-to-digital converter).

To measure the resistance, a dedicated circuit with a current-to-voltage converter (i.e., transimpedance amplifier) was used. The schematic diagram of this circuit is presented in Figure 1. The converter is based on the operational amplifier *OA*, the programmable reference voltage source *V_REF_* and the programmable resistor *R_FB_*, in a feedback loop. *R_X_* is the unknown skin resistance being measured. Switches *SW*_1_ and *SW*_2_ enable to inverse the polarity of the voltage applied, during the measurements at the pair of electrodes, which prevents the application of a constant voltage to the skin, during the consecutive measurements. The switch *SW*_3_ is used to measure the voltages *V_IN_* and *V_OUT_*, using the same analog-to-digital (A/D) converter *ADC*. First, the switch *SW*_3_ is set to the position 1 and 800 analog-to-digital conversions are made, and the average value from those 800 conversions is calculated and saved as the voltage *V_IN_*_1_. The same procedure is performed for the switch in position 2, to obtain the voltage *V_IN_*_2_, and for the switch in position 3, to obtain the voltage *V_OUT_*. The voltage *V_IN_* is then calculated as *V_IN_* = |*V_IN_*_1_ − *V_IN_*_2_|. Thus, to measure the resistance, there are 3 × 800 = 2400 A/D conversions that last for 360 ms, in total.

Assuming ideal properties of *OA* amplifier, the measured resistance *R_X_* can be calculated from the following set of equations:

(1) $$\left\{ \begin{array}{l} V_{OUT}=V_{REF}-I_{IN}R_{FB}\\ V_{IN}=-I_{IN}R_{X} \end{array}\right.$$

As,

(2) $$R_{X}=\frac{R_{FB}V_{IN}}{V_{OUT}-V_{REF}}$$

where *V_IN_* and *I_IN_* are the input voltage and current, respectively, *V_OUT_* is the output voltage of the transimpedance amplifier. As can be seen from Equation 2, changing the value of the resistor *R_FB_* and/or the voltage *V_REF_* enables measurement of the resistance, in a wide range. The resistance *R_FB_* was realized as a programmable resistance consisting of three switched discrete resistors: *R_FB_*_1_ = 10 kΩ, *R_FB_*_2_ = 100 kΩ, and *R_FB_*_3_ = 1 MΩ. The reference voltage *V_REF_* is obtained from the digital-to-analog (D/A) converter. The *V_REF_* voltages in the range 0.2 V–2.55 V are used at 3.3 V power supply. The circuit is controlled by the microcontroller, which is responsible for interfacing with A/D converter, switching the measuring electrodes, adjusting the programmable values of the *R_FB_* resistor, setting the voltage source *V_REF_* by programming the D/A converter and controlling the input signal of A/D converter with switch SW_3_.

**Figure 2:**
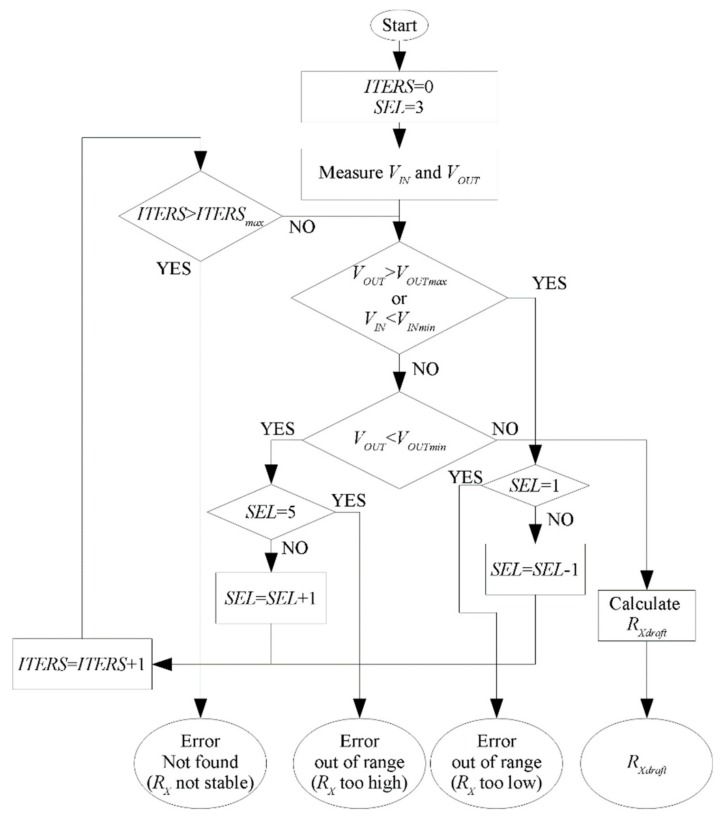
The algorithm used in the measurement of *R_Xdraft_*. The values of *SEL* represent the preselected sets of the *R_FB_* and *V_REF_* for draft measurement and they are explained in Table 1, *V_INmin_* = 25 mV, *V_OUTmax_* = 3.1 V, *ITERS_max_* = 3.

The circuit has been designed to measure *R_X_* in the range of, approximately, 1 kΩ–12 MΩ. To obtain the smallest measurement error, for each value of *R_X_*, the values of the *R_FB_* and *V_REF_* should be optimally adjusted. However, to calculate the optimal values of *R_FB_* and *V_REF_*, the unknown measured resistance *R_X_* must be known a priori. To overcome this problem, the measurement is performed using a two-stage approach. First, a draft measurement of the resistance *R_Xdraft_* is made, according to the algorithm shown in Figure 2 (*R_Xdraft_* is used to estimate the optimal values of *V_REF_* and *R_FB_*), then the final measurement of *R_X_* is made, using near optimal values of *V_REF_* and *R_FB_*.

For measuring *R_Xdraft_*, a set of a few values of *V_REF_* and *R_FB_* has been chosen, as shown in Table 1, which are selected by the variable *SEL* to cover the whole range of the measured resistance, using only five possible settings. As can be seen in Figure 2, if the value of the resistance *R_X_* is out of the measurement range, an error is returned instead of the resistance value. When the contact to the skin is not stable and the resistance varies quickly, during a single measurement of *R_Xdraft_*, the value of *ITERS* can be exceeded and the algorithm can end up with “not found” error. These errors are only reported to the user and they are not taken into account when analyzing stress.

Using the result of the draft measurement *R_Xdraft_*, the values of *R_FB_* and *V_REF_* are calculated. To obtain high accuracy of the A/D conversion, the measuring circuit has to provide, as high as possible, the levels of both *V_IN_* and *V_OUT_*. From Equation 1 it can be inferred that *R_FB_* must be as close as possible to *R_X_* (ideally *R_X_* = *R_FB_*) and *V_REF_* should be calculated, assuming the target level of *V_OUT_* is denoted as *V_OUTtarget_* as:

(3) $$V_{REF}=\frac{V_{OUTt\arg et}}{1+\frac{R_{FB}}{R_{Xdraft}}}$$

In this way, the final measurement of *R_X_* is performed in the conditions very close to optimal. The system is configured to report skin resistance, every 2 s.

### 2.2. The Wireless Transmission of the Results Using Bluetooth Low Energy

**Figure 3:**
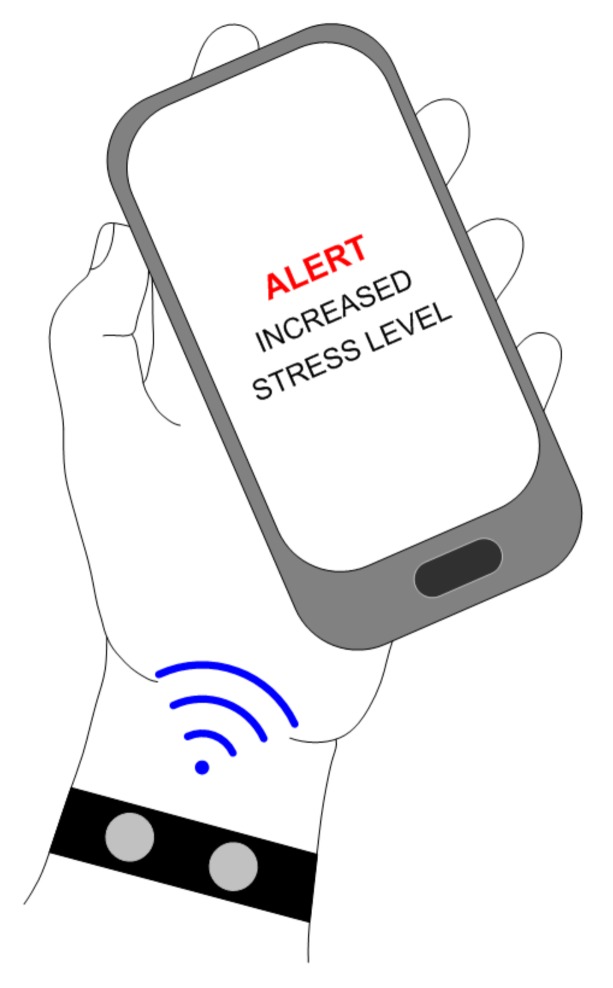
Exemplification of stress level increase alerting by a smartphone application.

The resistance measurement results are saved in an on-chip memory and they can also be broadcasted continuously. In the proposed system, a Bluetooth Low Energy (BLE) on-chip block was used as a communication device. In the BLE specification [53], there are different kind of roles a device can be in Peripheral, Central, Server, and Client role. The Peripheral role and the Central role define the possibility of the device to initiate a link between devices (only the device in a Central role can request to establish a connection); the device in the Peripheral role is usually constrained in resources, such as energy and computing power. The Server role denotes the device that contains data, while the Client role–the device that can read data. There is no connection between these roles, it is common for a Peripheral to be a Server, but other combinations are also possible. The BLE block used in this project has been configured to perform the role of a Peripheral device, as well as the role of a Server. For the purpose of this solution, a custom data format for the transmission of the measured resistance values was used. Such a custom data format is called “custom characteristics”, in the BLE standard [53]. The skin resistance values measured by the proposed system can be received by any BLE client, with the support of the custom BLE service, for example, an application running on a smartphone. In this way, although the dedicated application is not already developed, the skin resistance can be easily graphically presented to a human as a stress level increase indicator, as is exemplified in Figure 3, or can also be notified by a sound or vibrations.

### 2.3. The Implementation of the System

**Figure 4:**
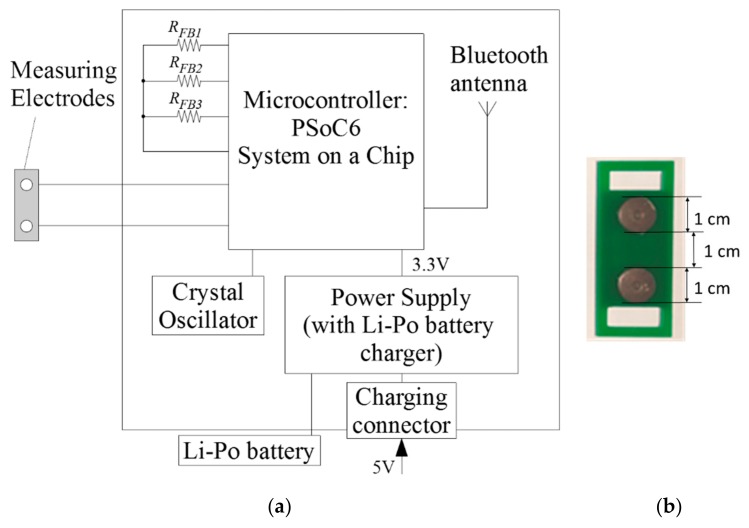
Measurement system: (**a**) Block diagram of the system with Cypress PSoC6; (**b**) a picture of the measuring electrodes.

The described system has been realized using programmable mixed-signal system-on-chip PSoC6, from Cypress. Most of the components of the presented system have been realized inside a single PSoC6 chip: The switches *SW*_1_–*SW*_3_, the switches of the programmable resistance *R_FB_*, the operational amplifier *OA* (OpampTIA component in PSoC6), the A/D converter ADC (Scan_ADC component in PSoC6), the D/A converter for generation of the voltage *V_REF_* (VDAC component in PSoC6), the BLE communication block and the microprocessor system with memory and peripherals. Only the *R_FB_* resistors and a small Bluetooth antenna (1 cm × 0.8 cm, realized on the copper layer of the printed circuit board) are off the chip. The block diagram of the system and a picture of the measuring electrodes have been shown in Figure 4a,b, respectively. The operation of the system is controlled by the custom application written in C language and running under a FreeRTOS operating system on ARM Cortex M4 processor inside a PSoC6 chip.

## 3. Materials and Method

Conformity of resistance levels, which validated the device, was tested using a commercially available system, consisting of a skin conductance sensor (0–30 µS ± 5%) connected to the analytical device (FlexComp Infinity by Thought Technology, Montreal-West, QC, Canada), which allowed for the gathering of data. The device was controlled by a computer with Thought Technology BioGraph Infiniti software. The system allowed for measurements of skin resistance, which was calculated as an inversion of the conductance within the software. To test the designed device, sixteen test subjects, between the ages of 15 to 50, of both genders (8 males and 8 females), participated in this study. The diversity of skin resistance, depending on the sensor placement, was measured and observed. The dry electrodes were fastened to the user’s hands by a strap and both connected to the dominant and non-dominant hand, and then the results for the dominant hand were chosen for further analysis.

**Figure 5:**
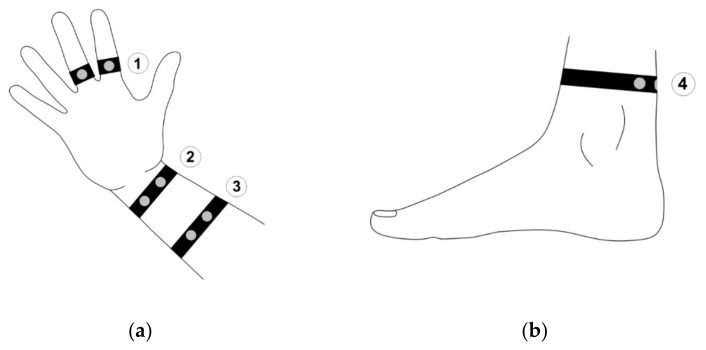
Placement of the sensors: (**a**) On the arm, (**b**) on the leg; 1—index and middle fingers (reference point), 2—wrist, 3—forearm, and 4—ankle.

Each participant was examined with a frequency of 2048 samples per second, for a 2 min per sensor placement. The first 50 s allowed to regulate physical response and they have not been included in the presented results. The skin resistance sensor was placed on the index and middle fingers to establish a baseline for each participant. These points provide accurate measurements which have been described by M. Jędrzejewska-Szczerska et al. [28]. A distribution of the sensors is illustrated in Figure 5 below.

For each subject, tests were performed during sensor placement on the wrist, forearm, and ankle. In all cases, the electrodes were separated by the length of the electrode’s diameter, in order to determine deviation from the reference point. This was an improvement, with respect to previous examinations by Villarejo et al. [47], in which the skin resistance was measured using a voltage source and a simple resistive divider with an A/D converter, which had a 12-bit resolution. For some skin resistance values, the measurements have a large error due to low levels of measured voltages. However, in this paper, the skin resistance was measured with an A/D converter with the same 12-bit resolution, but an active analog current-to-voltage converter has been used to feed the signals to the A/D converter. The current-to-voltage converter has been realized as a transimpedance amplifier, with the reference voltage and the programmable feedback resistance both adjusted to the optimal values, for each measurement, to provide the best possible signal levels.

## 4. Research and Results

Data gathered from the developed system, which is presented in Table 2, has similar resistance levels to the commercially available system, which confirms that the system was designed correctly.

The next step was the investigation that allowed the authors to determine in which sensor placement the skin resistance could be replicated, most accurately, in comparison to the reference point. Values of skin resistance gathered from each participant, in every measured point, are shown in Table 3.

**Figure 6:**
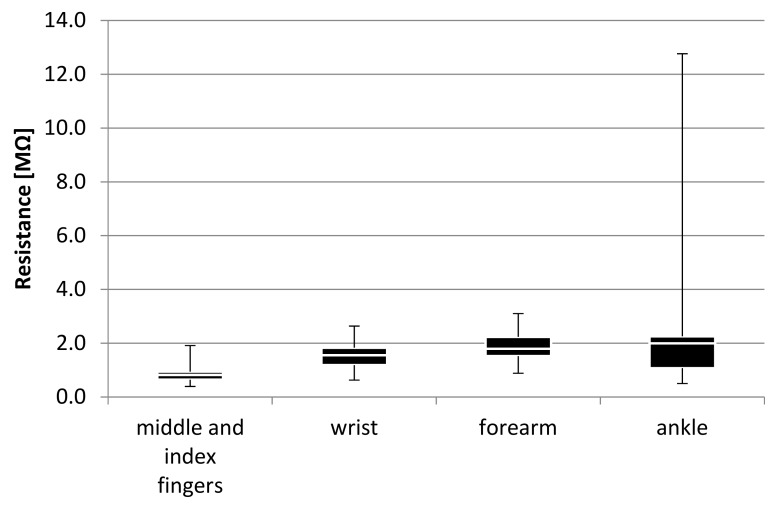
Statistical analysis of the results depending on sensor placement.

The lowest value of skin resistance was observed when the sensor was placed on the middle and index fingers, which was consistent with the initial assumption, therefore confirming the choice of this point as a reference. The data gathered from the middle and the index fingers also showed the smallest range of the values, which was a result of highest sensitivity to external stimuli in this sensor placement. The data collected on the wrist and forearm were comparable. However, the one on the forearm had the narrowest distribution, indicating a higher repeatability. Values obtained on the ankle varied the most. Graphical representation of the results, shown in Figure 6, also supported such reasoning.

Figure 6 shows the highest distribution, when the sensor was placed on the ankle, suggesting that the measurements in this point were the least stable, therefore, it should be excluded from further consideration. The lowest distribution of the data measured in the middle and index fingers affirmed this point as a reference. Distributions of data from wrist and forearm placements were similar. However, resistance levels between them suggested more accurate measurements on the wrist.

## 5. Discussion

The preliminary study allowed us to design the sensor network, which would, in future, be able to support adults with ASD, at the workplace, but it needs to be tested further in a real working environment on people with ASD.

Of course, there is also a need for complementary usage of other sensing methods in order to make stress detection more accurate. As a result of expanding the range of measurements by using other types of sensors based on various human body parameters, the support for employees with ASD, in the workplace, can become more effective. For improvement of stress level monitoring in the future, we plan to add other sensing methods to the wrist-worn device presented above. There is a possibility to implement other types of sensors, similar to those which were presented above in the literature overview, e.g., sensors to read body temperature, pulse, and body movement. Ultimately, readings from the sensors could be connected to a network of sensors recording various types of physical parameters e.g., brightness, humidity, or temperature [54,55] and controllers of its values. As a consequence, a type of “smart home” [56], a “smart workplace” concept could be developed. Many of the distracting factors, such as noise or brightness, which influence the stress levels of people with ASD, could be strongly limited or eliminated and as a result, increase the work comfort of such people.

The sensor network development outlined above can create new opportunities for ASD workers. Previously, some of these sensors were exploited commercially for the general population, e.g., in sports wristbands, smart watches, for supporting diagnosis and fostering therapy practices, for children with ASD. The solution presented above and a draft of a possible network of sensors development has been addressed for a certain group—adults with ASD. It is important because, over the last twenty years, the number of children diagnosed with ASD has increased significantly. Some estimations suggest that one out of every sixty-eight children in the United States may be affected by this disorder. As a result, over the next eight years, a 230% increase has been predicted [57] in the number of young people with ASD who will be transitioning to adulthood and potentially enter the labor market. Therefore, there is no need to prove the strong necessity of expanding the employment opportunities for them, because as was mentioned before, there are only some companies that focus on recruiting neurodiverse employees [6].

Creating a better work environment for this group can be a factor that will allow such people to foster dealing with their limitations and take advantage of their strengths, consequently, allowing them to achieve and maintain successful employment in some work roles, such as software testers or data analysts, and changing their unfavorable situation. There is a strong need to continue investigations in order to develop and implement appropriate solutions for people with ASD.

## 6. Conclusions

The main contribution of this work was to design and develop a tailored solution for assisting people with ASD, in their day-to-day work, in order to integrate them into the workforce. A Bluetooth skin resistance sensor design and construction were discussed. Additionally, trial tests on a group of sixteen people were performed to verify that the device functioned correctly. Various locations were used in order to make comparisons and allow for the selection of the most appropriate placement of the device. Statistical analysis of the data and convenience of the wearer allowed us to select the wrist as the most appropriate placement of the sensor. Considering the purpose of the assisting device, which was aimed at increasing the work performance of people with ASD in the workplace, the wrist was much more convenient and more discreet a location, than the fingers. As it was proposed, by using a Bluetooth Low Energy block, users could be notified immediately about their abnormal stress levels via a dedicated smartphone application.

The novelty of this solution is based on a number of attributes which, according to the best knowledge of the authors, did not occur yet in previous examinations. Firstly, it differs from previous solutions because of its special character bound up with a unique target group. It is aimed to be narrowly tailored and addressed to users with very specific needs—adults with ASD. Secondly, it also takes into account the specific context, which had been ignored before—the labor activity of those people. Assisting-solutions that support the increase in performance level for employees with ASD, in the work environment, is a completely new concept. Furthermore, another difference of the proposed device from other GSR devices (mentioned above and available in the market) is the idea of perceiving the sensing device utility, from a broader perspective, as a possible component of a network of sensors within a future “smart workplace”. Finally, the solution presented in this paper will allow the subject to be notified of a stress increase in real-time which has not been available so far by other GSR-sensing devices in the market, e.g., the Empatica E4 wristband [27]. The implementation of such a solution can aid, both, the people with ASD and those who work with them, in order to facilitate stress control and provide necessary adjustments to their workplace environment.

The described solution is addressed to well-functioning adults with ASD to support them in the workplace, so we assume that they will be able to consciously decide to use this assisting technology. In the authors’ opinion, the perspective of an improvement in their work performance and as a result, their labor market situation, is sufficient motivation for this group to use the proposed wearable device. Moreover, the wristband is convenient and a discreet means of locating the sensors.

## Figures and Tables

**Table 1:** The sets of the values of *R_FB_* and *V_REF_* used in the measurements of *R_Xdraft_*. The set used in the measurements is selected with the variable *SEL* in the software, according to the algorithm presented in Figure 2.

*SEL*	*R_FB_* [Ω]	*V_REF_* [V]	*R_X_* Range [Ω]
1	10 k	0.2	690–10 k
2	100 k	0.2	6.9 k–100 k
3	1 M	0.2	69 k–1 M
4	1 M	1.375	797 k–6.86 M
5	1 M	2.55	4.64 M–12.75 M

**Table 2:** Comparison between data gathered from the commercially available system (FlexComp Infinity by Thought Technology) vs. data gathered from the system presented in this paper.

Resistance [MΩ]	Minimum	Quartile 1	Median	Quartile 3	Maximum	Mean
(FlexComp Infinity by Thought Technology, Montreal-West, QC, Canada)	0.55	0.64	0.85	1.18	1.85	0.96
System presented in the paper	0.36	0.60	0.86	1.00	1.50	0.85

**Table 3:** Statistical analysis of skin resistance depending on the sensor placement.

Resistance [MΩ]	Minimum	Quartile 1	Median	Quartile 3	Maximum	Mean
index and middle finger	0.392	0.646	0.821	0.943	1.912	0.898
wrist	0.628	1.186	1.553	1.832	2.637	1.536
forearm	0.882	1.521	1.795	2.237	3.102	1.849
ankle	0.503	1.074	1.993	2.255	12.762	2.291
